# Active acoustic telemetry tracking and tri-axial accelerometers reveal fine-scale movement strategies of a non-obligate ram ventilator

**DOI:** 10.1186/s40462-020-0191-3

**Published:** 2020-02-10

**Authors:** Emily N. Meese, Christopher G. Lowe

**Affiliations:** 1 Department of Biological Sciences, CSULB Shark Lab, Long Beach, CA 90840 USA; 2 Present address: Texas A&M University Galveston, 1001 Texas Clipper Road, Galveston, TX 77551 USA

**Keywords:** Horn shark, Area restricted search, Activity patterns, Behavioral thermoregulation

## Abstract

**Background:**

California horn sharks (*Heterodontus francisci*) are nocturnally active, non-obligate ram ventilating sharks in rocky reef habitats that play an important ecological role in regulating invertebrate communities. We predicted horn sharks would use an area restricted search (ARS) movement strategy to locate dense resource patches while minimizing energetic costs of travel and nighttime activity. As ectotherms, we predicted environmental temperature would play a significant role in driving movement and activity patterns.

**Methods:**

Continuous active acoustic tracking methods and acceleration data loggers were used to quantify the diel fine-scale spatial movements and activity patterns of horn sharks. First passage time was used to identify the scale and locations of patches indicative of ARS. Activity was assessed using overall dynamic body acceleration (ODBA) as a proxy for energy expenditure. Behavior within a patch was characterized into three activity patterns: resting, episodic burst activity, and moderate, consistent activity.

**Results:**

After resting in daytime shelters, individuals travelled to multiple reefs throughout the night, traversing through depths of 2–112 m and temperatures of 10.0–23.8 °C. All sharks exhibited area restricted search patch use and arrived at their first patch approximately 3.4 ± 2.2 h (mean ± SD) after sunset. Sharks exhibited moderate, consistent activity in 54% of the patches used, episodic burst activity in 33%, and few (13%) were identified as resting at night. ODBA peaked while sharks were swimming through relatively deeper (~ 30 m), colder channels when traversing from one patch to the next. There was no consistent pattern between ODBA and temperature.

**Conclusions:**

We provide one of the largest fine-scale, high-resolution paired data sets for an elasmobranch movement ecology study. Horn sharks exhibited ARS movement patterns for various activity patterns. Individuals likely travel to reefs known to have profitable and predictable patches, potentially tolerating less suitable environmental temperatures. We demonstrate how gathering high-resolution information on the movement decisions of a community resident enhances knowledge of community structure and overall ecosystem function.

## Background

Mobile animals across all taxa make daily movement decisions to optimize daily energetic requirements. Since animals often live in environments that experience heterogeneous conditions with patchily distributed resources, they may modify their daily movements and behaviors in response to prey availability, environmental conditions, and their exposure to predation risk [1–4]. Therefore, animals display a variety of movement paths (i.e., search modes) to locate resource patches while minimizing movement and activity costs [2, 5, 6]. Many behavioral search modes have been proposed through the analysis of movement paths for a variety of taxa, such as Levy flights, Brownian walks, and area restricted search (ARS) [5, 7–10]. Here, we focus on ARS and define a patch as an area with an increased concentration of resources such as prey, shelters, or potential mates [11].

ARS has been demonstrated across a wide range of taxa of varying body sizes including insects [12, 13], birds [6, 14] and vertebrates [11, 15, 16] and is typically used to demonstrate foraging behaviors. When profitable, ARS allows individuals to maximize resource acquisition since the probability of encountering additional resources in the immediate vicinity (i.e., patch) is high [11, 17]. Therefore, when a resource patch has been identified, an individual will reduce its overall speed and may begin to display highly tortuous movements [10–12, 15, 18, 19]. However, the probability of foraging success decreases as predators remain in a patch, as mobile prey can become aware of the forager and leave the patch themselves or use crypsis strategies. Once the forager leaves the patch, prey can repopulate, thereby demonstrating the benefit of foragers to efficiently navigate back to the patch at a later time to re-evaluate prey abundances [20]. This can be particularly beneficial during times of reduced prey availability and poor environmental conditions in areas other than the patch [6].

Marine predators, such as coastal elasmobranchs (i.e., sharks and rays), exhibit a wide variety of movement patterns and behavioral strategies in order to maximize fitness over several spatial (e.g., meters to kilometers) and temporal (e.g., daily, seasonal) scales [21–24]. Non-obligate ram ventilating elasmobranchs are unique as they can rest on the seafloor and remain there for hours, up to a full day [25–27]. Therefore, resting elasmobranchs likely have strategies to manage tradeoffs associated with residing in an area, such as maintaining prey resources, staying vigilant to predators, and potentially being more tolerant of changing environmental conditions.

As ectotherms, water temperature is known to be a key abiotic factor that influences elasmobranch physiology and movement patterns [28–31]. The degree of metabolic temperature sensitivity (metabolic Q_10_) of an ectotherm will determine the extent of how dynamic thermal conditions may allow them to remain in their current environment or cause them to move and seek out more suitable thermal conditions [29, 32–34]. Thermally insensitive species may be able to select and reside in environments that others cannot, while thermally sensitive species may exhibit behavioral thermoregulation by moving through heterogeneous thermal habitats to maintain body temperature within a preferred range [32, 35]. Alternatively, some elasmobranchs have demonstrated behavioral thermoregulation through “shuttling” (e.g., “hunt warm – rest cool”) between opposing thermal environments to increase digestive efficiency and maximize energetic gain [32, 36–38]. Therefore, when quantifying movement patterns of ectotherms, particularly in the marine environment, it is important to consider dynamic thermal environments within their home range [25, 32, 38].

Measuring animal movements in a marine environment is typically done with acoustic or satellite telemetry [39–42]. Although these methods gather data on the geo-location and movement path of an individual, they often lack sufficient spatial and temporal resolution needed to classify fine-scale behaviors (i.e., resting, fine-scale restricted area searching, foraging) as well as the potential drivers of daily activity patterns [43]. Acceleration data loggers (ADLs) have been incorporated into tagging methods to measure individual behavior while complementing spatial movement data [25, 44]. Typically, ADLs include an accelerometer that can measure acceleration in three dimensions (i.e., heave, surge, sway [g]), as well as the capability to record environmental variables such as depth, temperature, and time of day [3, 45–47]. Therefore, combining spatial movement data with ADL data can geo-reference finer-scale activities and provide environmental context to movement and activity patterns. Ultimately, this results in a spatial representation of the animal’s energetic costs (i.e., energetic landscape) which can be used to identify profitable areas to an animal [21, 48–50].

In the present study, California horn sharks (*Heterodontus francisci*) were used as a model species to represent non-obligate ram ventilating elasmobranchs. California horn sharks (herein “horn sharks”) are small (< 1 m), nocturnally active, and relatively abundant residents in temperate Northeast Pacific rocky reef kelp beds [51, 52]. Horn sharks are considered to serve an important ecological role in regulating patchily distributed invertebrates as they prey on squid, crabs, urchins, and occasionally small fishes [53, 54]. Until now, fine-scale movements of horn sharks and the mechanisms that drive their movements and activity patterns have been largely unexplored. Therefore, the goal of this study was to quantify the diel, fine-scale spatial movements and activity patterns of horn sharks by combining continuous active tracking with high-resolution, three-dimensional (3D) acceleration data to better understand their ecological role in rocky reef communities. Specifically, we predicted that these demersal sharks exhibit ARS to locate resource patches and maximize energetic gain. Additionally, we predicted that water temperature would play a significant role in driving horn shark movement and activity patterns.

## Methods

### Field data collection

**Fig. 1:**
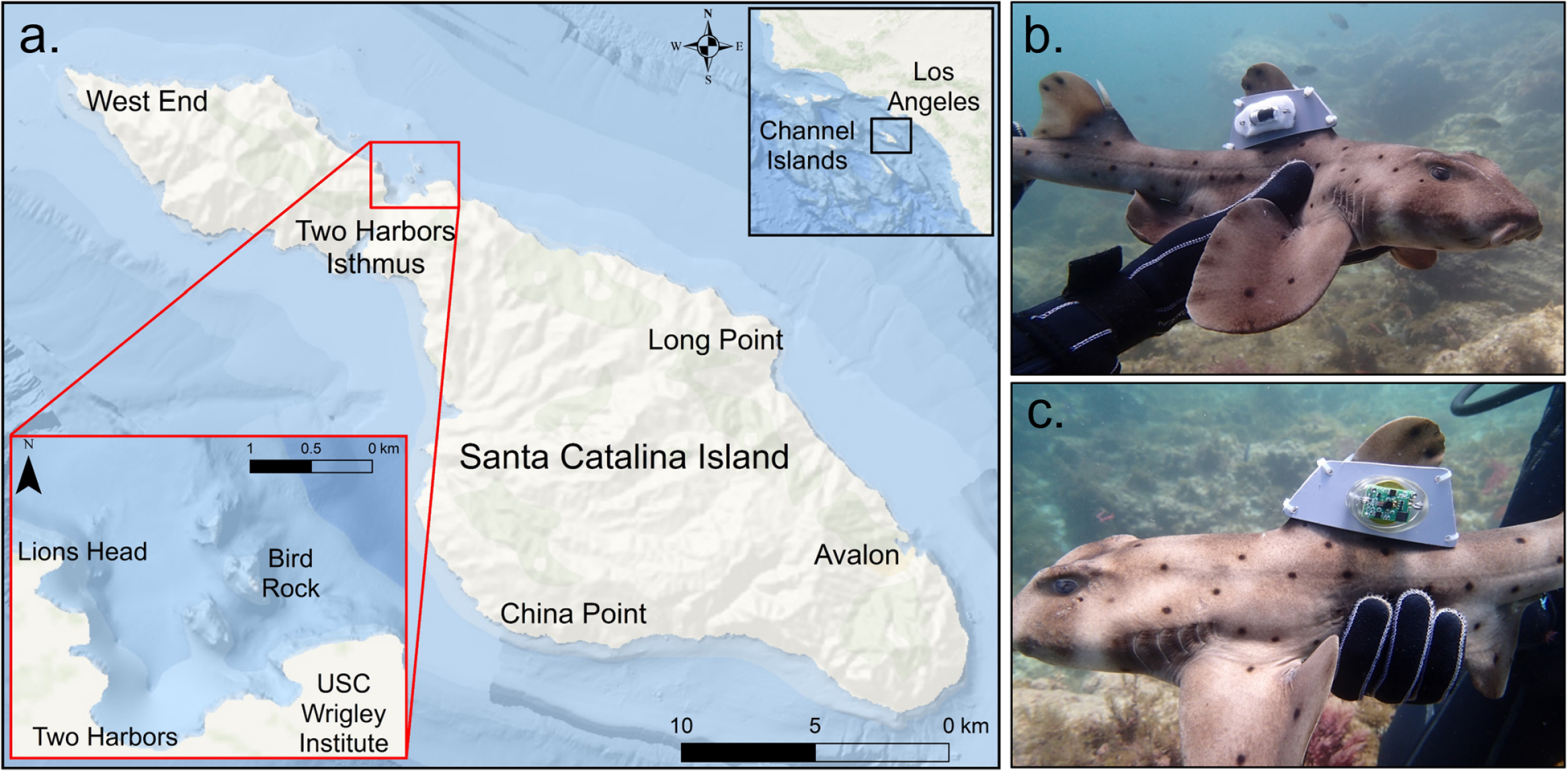
Study location (**a**) at the isthmus of Santa Catalina Island, California and custom tag package including a continuous acoustic transmitter (**b**) and an acceleration data logger (**c**)

To quantify fine-scale movements and activity patterns, California horn sharks (*n* = 21) were continuously actively tracked using acoustic telemetry at Santa Catalina Island, California (Fig. 1a). Individuals were fitted with custom-built dorsal fin tag packages which included a continuous-pulse acoustic transmitter (Vemco, V9-6x-1 L, 21 mm long × 9 mm diameter, 3.6 g in air, 2.1 g in water, power output 145 dB, pulse rate 2000 ms, 75 or 78 kHz) and an acceleration data logger (ADL) (Cefas G6a^+^ (*n* = 9): 40 mm × 28 mm × 17 mm, 19.5 g in air, 5.2 g in seawater; or TechnoSmArt (*n* = 12) Axy-Depth: 12 mm × 31 mm × 11 mm, 6.5 g in air) (Fig. 1b, c). Two ADLs were used during this study because they differed in battery life capabilities, and after being calibrated for accuracy they showed no difference in acceleration output. To tag individuals, divers would locate and capture a resting shark based on pre-existing knowledge of local distributions [52]. Underwater, we measured total length (TL, cm), girth (cm) and recorded sex, then attached the tag package, and released sharks back into their daytime shelter. After capture and tagging, each shark was continuously, actively tracked from a surface vessel for up to 48 h using a gunwale-mounted directional hydrophone (VH110, Vemco) and acoustic receiver (VR100, Vemco) [55–57]. As tagged sharks were actively followed, the VR100 receiver recorded the tracking vessel’s geo-position every time a transmitter pulse was detected. Based on range tests conducted at the study location, positional accuracy was determined to be 5–10 m at a gain of 0 dB and signal strengths between 80 and 105 dB, but varied with water depth, substratum type, and sea state. For data analysis, the tracking vessel’s geo-position was assumed to equal the shark’s position. At the end of the tracking period (at least 1 h after sunrise), divers would relocate the shark with an underwater acoustic receiver (RJE International DPR), remove the tag package to download the ADL, and fin notch individuals to eliminate likelihood of double sampling.

### Data analyses

All mapping and geo-referenced analyses were performed in ArcGIS (vers. 10.4) (ESRI). Modelling and statistical analyses were done in R (vers. 3.5.2) (R Foundation for Statistical Computing). For most analyses, data were analyzed in 24 h diel cycles, separated into daytime and nighttime periods using the *suncalc* package in R to determine local sunrise and local sunset times for each tracking day [58].

### Spatial movements

For spatial movement analyses, geo-positions derived from active tracking were filtered to only include detections of highest positional accuracy (gain 0 dB, signal strengths 80–105 dB). To estimate two-dimensional (2D) activity space for each individual, Brownian bridge kernel utilization distributions (BBKUDs) were calculated in R with the *adehabitatHR* package [59]. By using BBKUDs, we were able to account for the time dependence between locations and estimate the area where the animal was most likely to be located [60]. The BBKUDs were calculated at a 95% area to estimate daily activity space, and a 50% area to estimate core activity space [61]. Daytime and nighttime BBKUDs were calculated for each individual, for each 24 h diel cycle. Linear mixed effects models (LMEs) using the lmer function from the *lme4* package in R [62, 63] were used to statistically compare total areas of daytime and nighttime BBKUDs (95 and 50% analyzed separately) and to statistically compare male and female nighttime activity space (95 and 50% analyzed separately), with individual included as a random effect. LMEs were selected to account for unequal sample sizes across categories (i.e., daytime versus nighttime and male versus female). To obtain associated *p*-values, we used the Anova function in the *car* package with Type II Wald Chi-square tests [64, 65].

To identify the scale and location of potential patches via area restricted search (ARS) patterns, a first passage time (FPT) analysis was performed using the *adehabitatLT* package in R [66]. The FPT analysis measures how much time it takes an individual to cross a circle of a given radius [11, 14, 15]. The time measured will vary depending on the size of the patch area as well as the individuals speed and path tortuosity within the patch [14, 67]. We only used nighttime movement paths to avoid detecting patches that were associated with daytime resting positions. Additionally, we used the nighttime 50% BBKUD for each individual to determine the maximum radii to search for periods of ARS (range of radii: 5–100 m) [11]. For the FPT analysis, we used the Lavielle path segmentation method [68] to identify how many segments (i.e., fragment of movement path) indicated ARS and where those segments occurred within the nighttime movement path. The Lavielle method was chosen because it estimates the most likely segmentation for a movement path built of K segments, by minimizing a penalized contrast function (i.e., the penalty on the number of model parameters and a weighting of that penalty) [67, 68]. We specified the minimum length of a segment to be 10 m and the maximum number of segments (K_max_) to be 20 to ensure all possible segments would be accounted. The ideal number of segments for each sharks’ trajectory (K_opt_) was determined by choosing the last value of K for which the second derivative D_(k)_ of the standardized contrast function was greater than the threshold of S = 0.75 [68]. For this study, K_opt_ varied from 2 to 7 segments across nighttime periods. Each Lavielle segment that indicated ARS was then imported into ArcGIS where they were visually assessed and minimum convex polygons (MCPs) were used to calculate the 2D area of the patch. Linear distances (km) travelled to and from a patch were measured in ArcGIS using 1) the centroid of the daytime resting shelter (obtained from the daytime 50% BBKUD) to the centroid of the ARS patch, 2) centroids in between ARS patches (if more than one patch was used for an individual), and 3) the distance from the centroid of the last ARS patch used to the next morning’s daytime shelter site. LME models were used to statistically test if there was a significant relationship between the time spent in a patch (response) and the area of a patch, distance travelled to get to a patch, or activity within a patch (% resting, see below). We conducted three separate LME models to determine how each predictor alone influenced time spent within a patch. Time spent in a patch (response) was the total time (min) spent within each patch and was calculated from the start and end times of patch use from the FPT analysis. To account for temporal autocorrelation associated with time spent, we included the number of patches per individual per night as another predictor in each model. Individual was included as a random effect, and *p*-values were obtained with the Anova function from the *car* package with Type II Wald Chi-square tests [64, 65]. To calculate Akaike Information Criterion (AIC) and deviance explained values, we refit the models using maximum likelihood (ML) instead of restricted maximum likelihood (REML). A null model was created to obtain a value for total deviance of the dataset. To calculate the deviance explained by each specific model, we used the following equation: [(total deviance from null model – total deviance from model)/total deviance from null model] [62].

### Activity patterns

ADLs recorded tri-axial acceleration data at 25 Hz and temperature and depth every 1 Hz (Cefas G6a^+^) or every 25 Hz (Technosmart AxyDepth). All acceleration data were processed in IgorPro (vers. 6.2, WaveMetrics) and analyses were conducted using Ethographer [69] and R. Static acceleration was separated from dynamic acceleration using a 2 s box smoother [70]. Overall dynamic body acceleration (ODBA) was calculated by summing the absolute value of the dynamic axes (i.e., heave, surge, sway) [46]. ODBA has been used as a proxy for activity across many taxa, including elasmobranchs, since body movements result directly from muscle contraction, using adenosine triphosphate (ATP) and therefore requiring oxygen [25, 46, 71–73]. Mean (± SD) and ranges of ODBA (g), temperature (°C), and depth (m) experienced by each individual for each 24 h diel cycle are shown in Additional file 1.

To classify each second of acceleration data as either active or inactive (i.e., resting), a spectrum analysis was generated using a continuous wavelet transformation with the Morlet wavelet function using the sway axis to quantify the periodicity and intensity of the acceleration signals [69, 74] (Additional file 2). A periodicity of 0.5–2 s was chosen based on the dynamic body acceleration observed for this species, and because it represents the range in length of time for one full tailbeat to be completed (based on observations made in captivity) [26, 69, 75]. By using the sway axis, the spectrum resulted in the same output when the animal was motionless (i.e., resting) even if its posture differed [26, 69]. With derived amplitude and frequency, every second from the spectrum analysis was grouped in a *k*-means cluster analysis to create 10 behavioral categories of the acceleration signals (see Additional file 2). We used a total of 10 clusters because it allowed the shapes of major spectra in the acceleration ethograms to become stable, while also ensuring we encompassed all behavioral patterns [25, 69]. By visually examining the signal strength amplitude and cycle of resulting clusters, we could visually interpret and assign a cluster as resting behavior because resting produced low-amplitude spectra, indicating the original behavior consisted of low acceleration and the lack of a clear cyclic signal [26, 76, 77]. The *k*-means clustering was performed on each individual rather than using the same classification values across all individuals because some individuals only displayed a single resting cluster whereas others displayed more than one [25, 26]. Using the identified resting clusters, every second of acceleration data was assigned a binary value for if that second exhibited resting or active behavior [25]. Binary activity values were used to calculate the mean (± SE) percent each individual spent resting per hour of day (24 h) for males and females.

In addition, binary activity values from the *k*-means analysis were used to estimate and classify activity patterns within ARS patches. Using the FPT analysis above (see Spatial Movements) times of patch use were acquired and used to find related periods of activity. For each identified patch, binary activity values were used to estimate the percent of time spent resting within a patch to first classify patches as resting (95% majority) or active. ODBA and depth within a patch were then visually assessed in IgorPro to further classify patch activity patterns. Resting patches were confirmed by observing low ODBA values (< 0.2 g) and no discernible changes in depth throughout the entire duration of patch activity. Active patches were identified by comparatively higher ODBA values (> 0.5 g), indicating periods of swimming tail beats or bursts of activity. From these assessments (*k*-means and visual), we identified three classifications of patch activity patterns including 1) resting (i.e., motionless for at least 95% of the time with no indication of other activity), 2) episodic burst activity (i.e., some time spent motionless with short durations of high ODBA [2–3 g]), and 3) active (i.e., consistent moderate to high values of ODBA [1–3 g]). Resting patches were removed from further patch statistical analyses because we could not confidently determine whether the shark was truly using a patch (i.e., remaining vigilant and either foraging or refuging from predators) yet spending a majority of the time motionless, or if the shark was instead inattentively refuging in a shelter.

To determine diel and environmental drivers in nighttime activity patterns (ODBA), we used general additive mixed models (GAMMs) using the *mgcv* package in R [78]. GAMMs were chosen because they allow for both the temporal autocorrelation and individual shark ID as a random effect to account for differences between individuals [25, 38, 47, 72, 79]. An auto-regressive process of order 1 (AR1) was used with elapsed time (nested within individuals) as the position variable to account for temporal autocorrelation in time series data [79]. The correlation at lag = 1, derived from an autocorrelation function (ACF), was included as a term in the AR1 function to specify the autocorrelation structure. GAMMs were constructed with a gaussian error distribution and all covariates were modelled using smooth splines. Since we were interested in understanding how diel and environmental factors influenced activity, GAMMs were analyzed for nighttime hours only for this nocturnal species. ODBA was analyzed over 5 min means to determine if there was a temporal pattern in nighttime activity. Temperature was separately analyzed over 5 min means to determine if there was a temporal pattern in nighttime temperature use. The relationship between ODBA and temperature during nighttime activity was then visually compared to determine if temperature was influencing nighttime activity patterns. ODBA was additionally analyzed with depth (5 min means) to determine if there was a relationship with activity and depth. Models were checked with the gam.check function in the *mgcv* package to ensure proper fit [78, 79].

## Results

### Active tracking and ADL summary

**Fig. 2:**
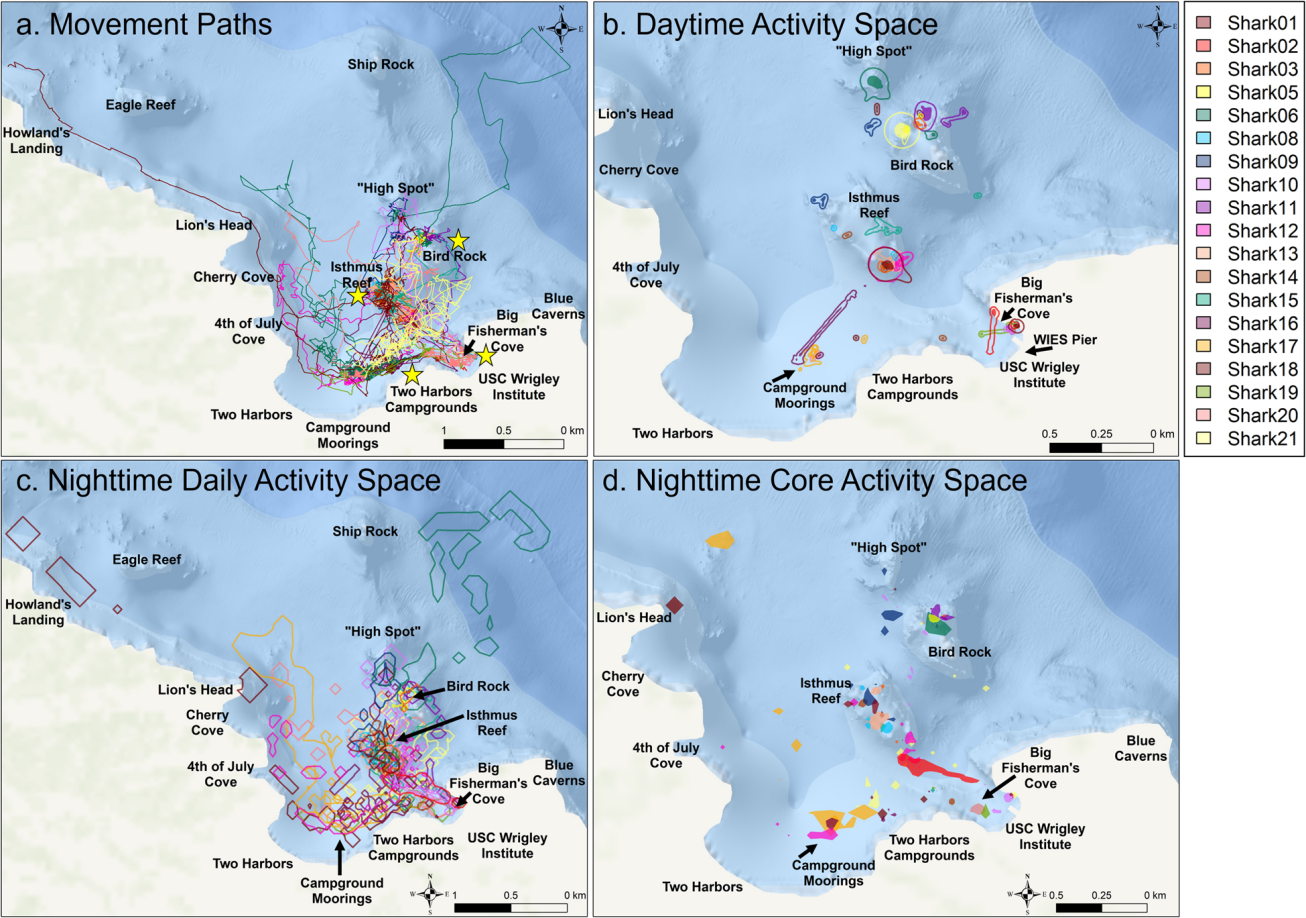
California horn shark (*n* = 19) active track movement paths (**a**) and activity space estimates for daytime (**b**) and nighttime (**c**, **d**). Individual horn sharks are color coded, with outlines indicating boundaries of daily activity space (95% BBKUDs) and solid polygons indicating core (50% BBKUD) activity space estimates. General tagging location reefs are identified with yellow stars in (**a**)

Twenty-one continuous active tracks were completed during Jun – Sept 2016 and Jun – Oct 2017 at Santa Catalina Island (Table 1, Fig. 2a). Sharks were tagged opportunistically at multiple rocky reef sites including Big Fisherman’s Cove (BFC; *n* = 8), Isthmus Reef (IR; *n* = 7), Bird Rock (BR; *n* = 3), and Campgrounds (CG; *n* = 3) (Fig. 2a). There were 13 females and eight males with a mean (± SD) length of 73.21 ± 6.25 cm (range: 59–89 cm; Table 1). The tag package fell off prematurely for Shark 04, so this shark was removed from all analyses. Shark 07 was lost during active tracking and the resulting spatial movement data that was acquired (< 5 h) was removed from analysis. However, we did recover the tag package and ADL data from Shark 07 approximately 2 weeks later, leaving 19 sharks for spatial movement analyses. The ADL malfunctioned for Sharks 02 and 15 allowing for activity analyses of 18 sharks (including Shark 07). Therefore, 17 sharks were used for analyses requiring paired spatial and activity data (Sharks 02, 04, 07, and 15 removed). During tag deployments, local sunrise varied from 05:42–07:07 (PST, median = 06:14), and local sunset times varied from 18:08–20:08 (median = 19:43).Geo-positions from active tracking data were geo-referenced in ArcGIS (Fig. 2a). All sharks remained within 3 km of their tagging location except for Shark 18 that travelled in a linear direction until sunrise and ended, approx. 3.9 km away from the tagging location in a single night (Fig. 2a). The average linear distance travelled (i.e., length of movement path) in a 24 h period (i.e., a single nighttime period) was (mean ± SD) 8.09 ± 3.21 km (range: 1.57–13.41 km). When divided into daytime and nighttime periods, there were 49 daytime periods (i.e., tagging day, middle day [for tracks > 24 h], and recovery day), and 30 nighttime periods (1–2 per individual depending on track duration).

**Table 1:** Active Tracking Summary for California horn sharks (*Heterodontus francisci*)

Shark ID	Length (cm)	Girth (cm)	Sex	Track Duration (h)	Dates	Acceleration Data Logger (ADL)	Tagging Location	Notes
Shark 01	79	32	M	24 hr	23-24 Jun 2016	Cefas G6a^+Cefas G6a+^	BFC	
Shark 02**	59	24	M	24 hr	11-12 Jul 2016	Cefas G6a^+Cefas G6a+^	BFC	ADL malfunctioned
Shark 03	70	27	F	24 hr	18-19 Jul 2016	Cefas G6a^+Cefas G6a+^	IR	
Shark 04 ***	76	34	F	13 hr	28-29 Jul 2016	Cefas G6a^+Cefas G6a+^	BR	Tag fell off prematurely
Shark 05	73	31	M	24 hr	01-02 Aug 2016	Cefas G6a^+Cefas G6a+^	BR	
Shark 06	76	36	F	24 hr	05-06 Aug 2016	Cefas G6a^+Cefas G6a+^	BR	
Shark 07 *	74	32	M	24 hr	08-09 Aug 2016	Cefas G6a^+Cefas G6a+^	IR	Lost after 5 h of tracking
Shark 08	61	23	M	24 hr	11-12 Aug 2016	Cefas G6a^+Cefas G6a+^	IR	
Shark 09	72.5	33	F	24 hr	02-03 Sept 2016	Cefas G6a^+Cefas G6a+^	IR	
Shark 10	71	34	F	48 hr	07-09 Jun 2017	TechnoSmArt Axy-Depth	BFC	
Shark 11	71	32	F	48 hr	14-16 Jun 2017	TechnoSmArt Axy-Depth	BFC	
Shark 12	66.5	26	F	48 hr	27-29 Jun 2017	TechnoSmArt Axy-Depth	IR	
Shark 13	77	35	M	48 hr	2-4 Aug 2017	TechnoSmArt Axy-Depth	IR	
Shark 14	70	31	F	48 hr	9-11 Aug 2017	TechnoSmArt Axy-Depth	IR	
Shark 15**	89	37.5	F	48 hr	16-18 Aug 2017	TechnoSmArt Axy-Depth	BFC	ADL malfunctioned
Shark 16	74	37	F	48 hr	23-25 Aug 2017	TechnoSmArt Axy-Depth	CG	
Shark 17	75	35	F	48 hr	28-30 Aug 2017	TechnoSmArt Axy-Depth	CG	
Shark 18	75	35	F	48 hr	11-13 Sept 2017	TechnoSmArt Axy-Depth	CG	
Shark 19	74	35	M	38 hr	2-3 Oct 2017	TechnoSmArt Axy-Depth	BFC	
Shark 20	78	33	M	48 hr	5-7 Oct 2017	TechnoSmArt Axy-Depth	BFC	
Shark 21	76.5	33	F	48 hr	23-25 Oct 2017	TechnoSmArt Axy-Depth	BFC	

Asterisks indicate sharks removed from analyses (* = removed from spatial analyses, ** = removed from acceleration analyses, *** = removed from all analyses). Tagging locations are abbreviated: *BFC* Big Fisherman’s Cove, *IR* Isthmus Reef, *BR* Bird Rock, *CG* Campgrounds

Sharks remained resting in a shelter throughout the day, then departed their daytime shelter at sunset. ADL data indicated that on average (mean ± SE), females spent 88.1 ± 1.3% of the day resting and 48.9 ± 3.1% of the night resting. Similarly, males spent 93.3 ± 2.2% of the day resting and 55.02 ± 3.47% of the night resting. We chose to not remove any acceleration data as most of our statistical analyses were performed on nighttime periods only. However, it is important to note the tagging day often had periods of increased ODBA (yet no indication of spatial movements) compared to subsequent daytime periods after the tagging day (i.e., middle and recovery days for tracks > 24 h). This increase in activity on the day of tagging was likely due to individuals being unaccustomed to the tag package. Since sharks were tagged during the morning, we assumed that by sunset, individuals had become accustomed to the tag package and nighttime activity was unaffected. This was further confirmed by no differences in activity patterns during the nights for individuals tagged for 48 h (i.e., two nighttime periods).

Sharks rested in depths 0.2–35 m (15 ± 1.4 m) during the day. As a shark remained resting in a shelter during the day, ambient water temperatures changed (i.e., daily tidal fluctuations) approximately 3.3 ± 0.3 °C around them and ranged from 11.0–23.0 °C (18.1 ± 2.3 °C). In contrast, sharks experienced a mean temperature range of approximately 7.4 ± 0.4 °C while traveling throughout the night. In addition, during the night, sharks travelled through depths ranging from 1 to 112 m (19 ± 15 m) and through temperatures from 10.0–23.8 °C (18.0 ± 3.5 °C; see Additional file 1).

### Diel activity spaces

Daytime BBKUDs ranged between 0.00028–0.0699 km^2^ (0.0048 ± 0.013 km^2^; 95% BBKUD) and 0.00006–0.0096 km^2^ (0.0008 ± 0.0019 km^2^; 50% BBKUD; Fig. 2b). Nighttime BBKUDs for horn sharks ranged greatly between 0.003–0.434 km^2^ (0.084 ± 0.092 km^2^; 95% BBKUD) and 0.00005–0.025 km^2^ (0.004 ± 0.005 km^2^; 50% BBKUD; Fig. 2c, d). Nighttime daily (95%) and core (50%) activity spaces were significantly greater than daytime daily and core activity spaces (LME 95%: *Chi-squared* = 36.39, *df* = 1, *p* = 0.0001; LME 50%: *Chi-squared* = 16.06, *df* = 1, *p* = 0.0001; Additional file 3). There was no difference in male and female activity space for either day or night for both daily and core activity spaces (LME 95%: *Chi-squared* = 1.67, *df* = 1, *p* = 0.195; LME 50%, *Chi-squared* = 0.019, *df* = 1, *p* = 0.888).

### Nighttime patch use

**Fig. 3:**
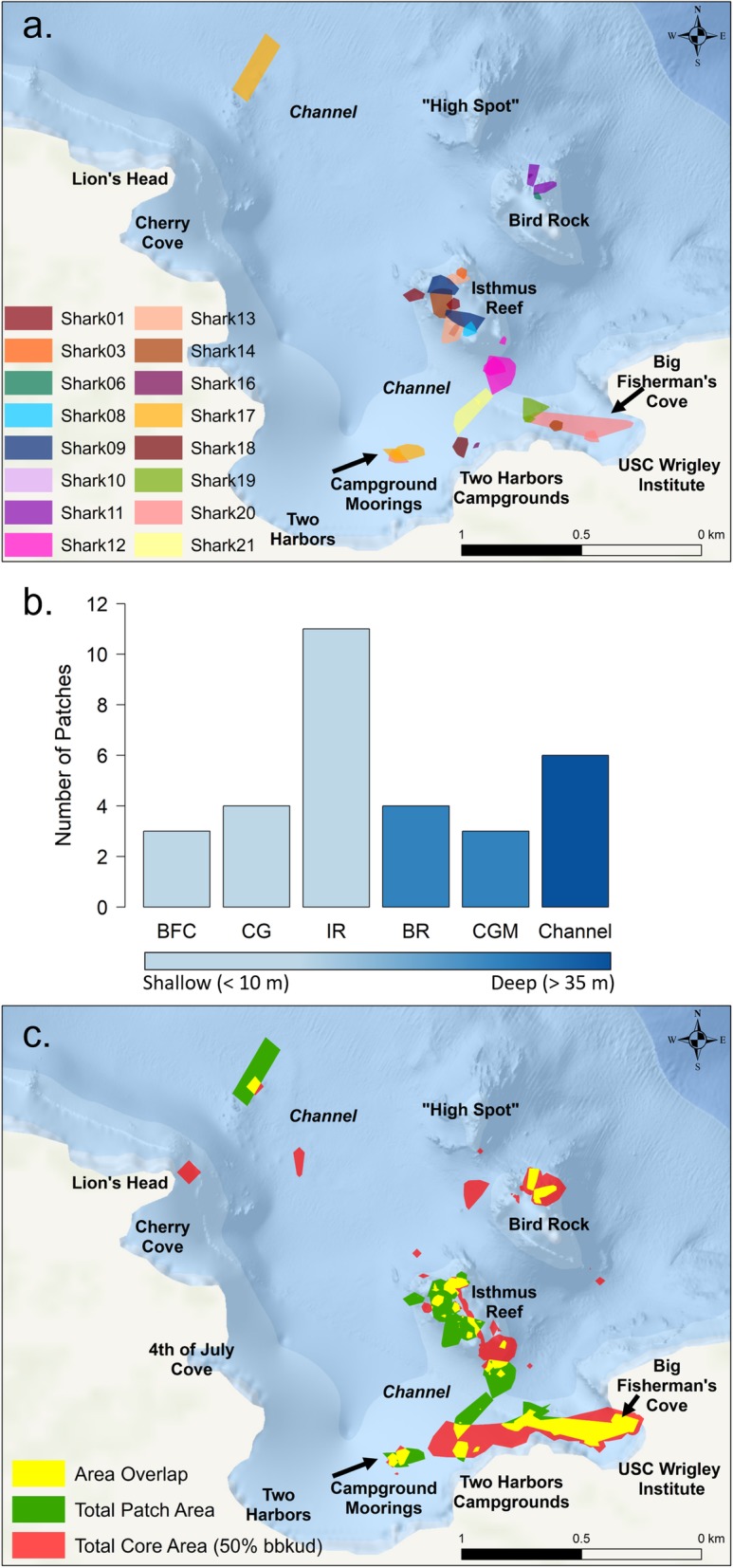
**a** Locations of area restricted search (ARS) patches (*n* = 31) color coded by individual shark (*n* = 16). **b** Patch frequency distribution by reef, colors represent average depth (m) per patch at each respective reef. Abbreviations denote sites in maps (**a**) and (**b**): BFC, Big Fisherman’s Cove; CG, Campgrounds; IR, Isthmus Reef; BR, Bird Rock; CGM, Campground Moorings. **c** Locations of area overlap (yellow) between total core (50% BBKUD) area (red) and total patch area (green)

**Fig. 4:**
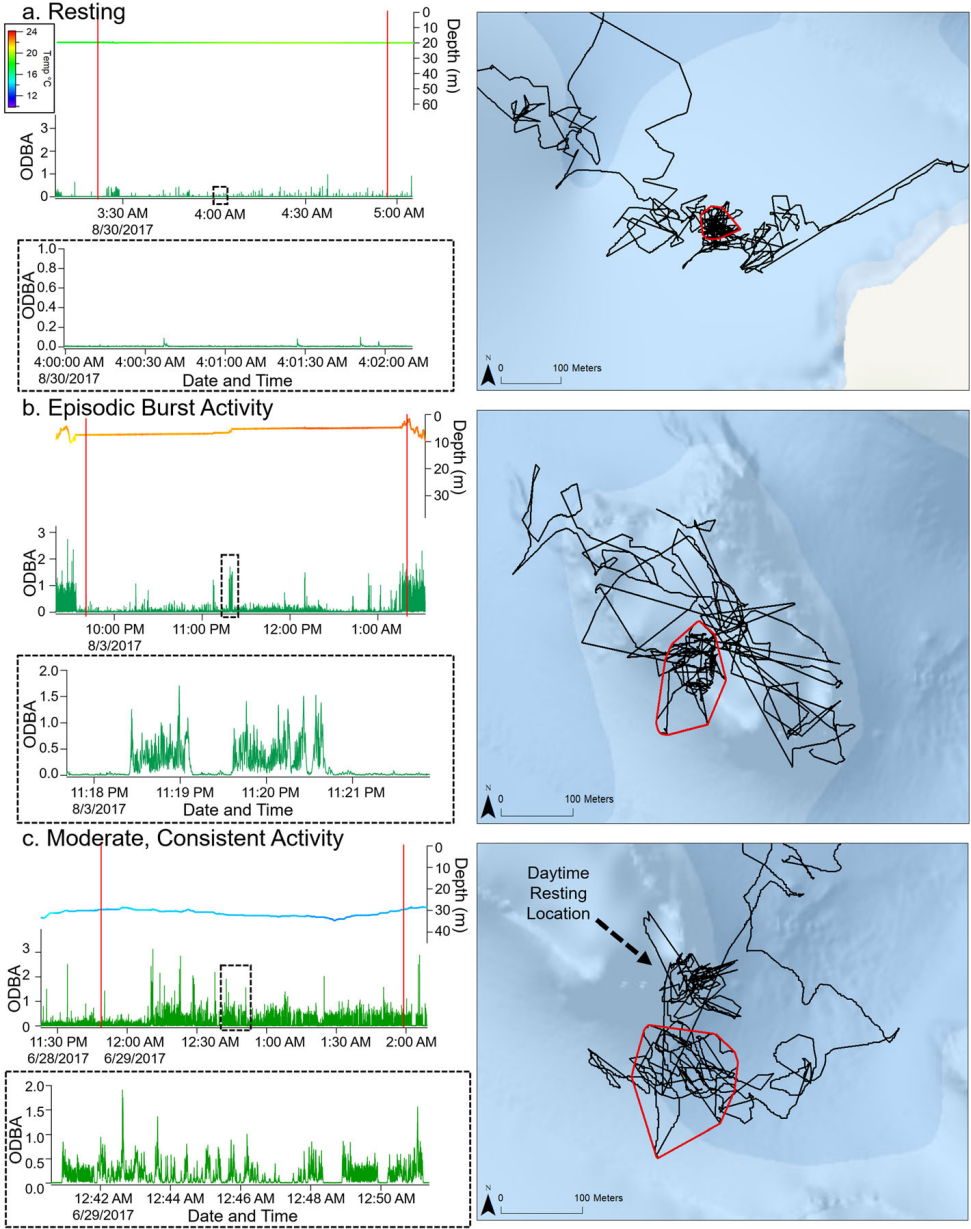
First passage time (FPT) analysis results showing area restricted search (ARS) patches for California horn sharks and corresponding acceleration signatures within each patch. Examples are of **a** resting, **b** episodic burst activity, and **c** moderate, consistent, activity. Acceleration signals include the depth trace colored by temperature, ODBA (green), and a zoomed in representation of the ODBA signal observed. Black traces on the maps are the movement paths. Red vertical lines denote the times within the patch circled in red on the maps. The part of the trace that looks like patch activity in the map in (**c**) is that individual’s daytime resting position, so it was not identified as a patch in the FPT analysis

A total of 38 patches were identified from the 27 nighttime periods analyzed (*n* = 17 sharks). Based on the results of the *k*-means binary activity values and the visual confirmation of ODBA, five patches (13%) were removed from further statistical analyses. Two additional patches were removed because their spatial characteristics were not consistent with our definition of a patch, leaving a total of 31 patches (*n* = 16 sharks) for further analysis (Fig. 3a). Of the remaining 31 active patches, 13 (42%) were identified to have episodic burst activity and 18 (58%) were identified to have moderate, consistent activity (Fig. 4). First passage time analysis identified patch radii of 34 ± 27 m (8–112 m). Area of patches (MCPs) ranged from 53 to 28,095 m^2^ (5447 ± 6533 m^2^) and the linear distance travelled to get to a patch ranged from 21 to 1747 m (450 ± 484 m). For most (79%) nighttime periods, a single patch was used, and only a few nights (21%) showed individuals used multiple patches (max: 3 patches per night).

All individuals arrived and departed patches around the same time of night. Sharks would arrive to the first patch of the night around 21:40 (PST, median), approximately 3.5 ± 2.4 h after sunset. Once an individual had found a patch, it spent 0.2–7.5 h (2.3 ± 2.0 h) in the patch. Individuals that used more than one patch throughout the night spent 2.0 ± 1.3 h travelling in between patches. All individuals left their last patch 3.5 ± 2.6 h before sunrise, at approximately 04:03 (PST, median) to then travel to their next daytime resting location. All sharks travelled from a final patch to a daytime resting shelter, so final patches were never the same as the final resting location. There was no significant relationship between the time spent in a patch and the distance travelled to get to a patch (LME: *Chi-squared* = 0.584, *df* = 1, *p* = 0.444, *dev*. = 3.79%), or the activity (% spent resting) in a patch (LME: *Chi-squared* = 0.165, *df* = 1, *p* = 0.685, *dev*. = 3.53%) (Table 2). However, as patch size increased, the time spent in a patch also increased (LME: *Chi-squared* = 13.349, *df* = 1, *p* = 0.0002, *dev*. = 10.18%) (Table 2). Number of patches per individual per night was significant for each LME model (Table 2).

**Table 2:** Linear mixed effects models (LMEs) to estimate predictors of time spent within a patch for California horn sharks (*Heterodontus francisci*)

Model Parameters									
Response	Predictors	Random	Likelihood	AIC	Total Deviance from Model	Deviance Explained (%)	*Chi-Squared*	Degrees of freedom	*p* value
Time in Patch	1 (null model)	(1|Track)	ML		141.05				
Time in Patch	Distance travelled to get to patch	(1|Track)	ML	145.7	135.7	3.79%	0.584	1	0.444
	Number of patches per individual per night						5.028	1	0.025
Time in Patch	Activity within patch (% of time spent resting)	(1|Track)	ML	146.1	136.1	3.53%	0.165	1	0.685
	Number of patches per individual per night						5.324	1	0.021
Time in Patch	Area of patch	(1|Track)	ML	136.7	126.7	10.18%	13.349	1	0.0002
	Number of patches per individual per night						7.446	1	0.006

LMEs were done in the *lmer* framework in R. Time in patch was calculated as the total time (min) spent in a patch and derived from the start and end times of patch use from a First Passage Time (FPT) analysis. Distance travelled to get to patch (m) and area of patch (m^2^) were measured in ArcGIS. Activity within patch (% of time spent resting) was calculated using the *k*-means analysis. Number of patches per individual per night was added to each model to account for temporal autocorrelation associated with time spent, and individual per night was included as a random factor. Maximum Likelihood (ML) was used to obtain AIC and deviance values. Percent deviance explained was calculated by subtracting the total deviance from the model from the null deviance and dividing by the null deviance. The AIC values are from the model summary and for each model independently, as no model comparison was made. Chi-squared values, degrees of freedom, and *p* values were obtained from the Anova function in the *car* package in R

Patches were identified on many reefs including BFC, BR, CG, CGM, IR, as well as the channel areas in between reefs. The reef with the most patches (35%) was IR (Fig. 3b). ADL data indicated that patch depth ranged between 3 and 60 m (18 ± 14 m), and there were clear site differences in depth distribution of patches (Fig. 3b). While IR is an isolated reef with shallow habitat (< 10 m) and sloping reef walls down to ~ 30 m, most patches occurred on the shallower rocky reef crest of IR (Fig. 3a, b). Patches that occurred in the channels were the deepest (36 ± 12 m). Mean water temperature in a patch across all individuals was 18.1 ± 3.03 °C (range: 12.1–22.3 °C), which did not differ from the mean temperature of nighttime activity (18.0 ± 3.5; see above and Additional file 1). To determine if patch use was driving core nighttime space use, we compared the resulting areas of the patches with the 50% BBKUD areas. When merged, patches encompassed a spatial area of 0.154 km^2^. In comparison, the total merged area of the 50% BBKUDs (that had associated patch use) was 0.182 km^2^. Therefore, the resulting area overlap of patch space to core activity space was 0.072 km^2^, which amounted to an 84.7% overlap (Fig. 3c).

### Nighttime activity patterns

**Fig. 5:**
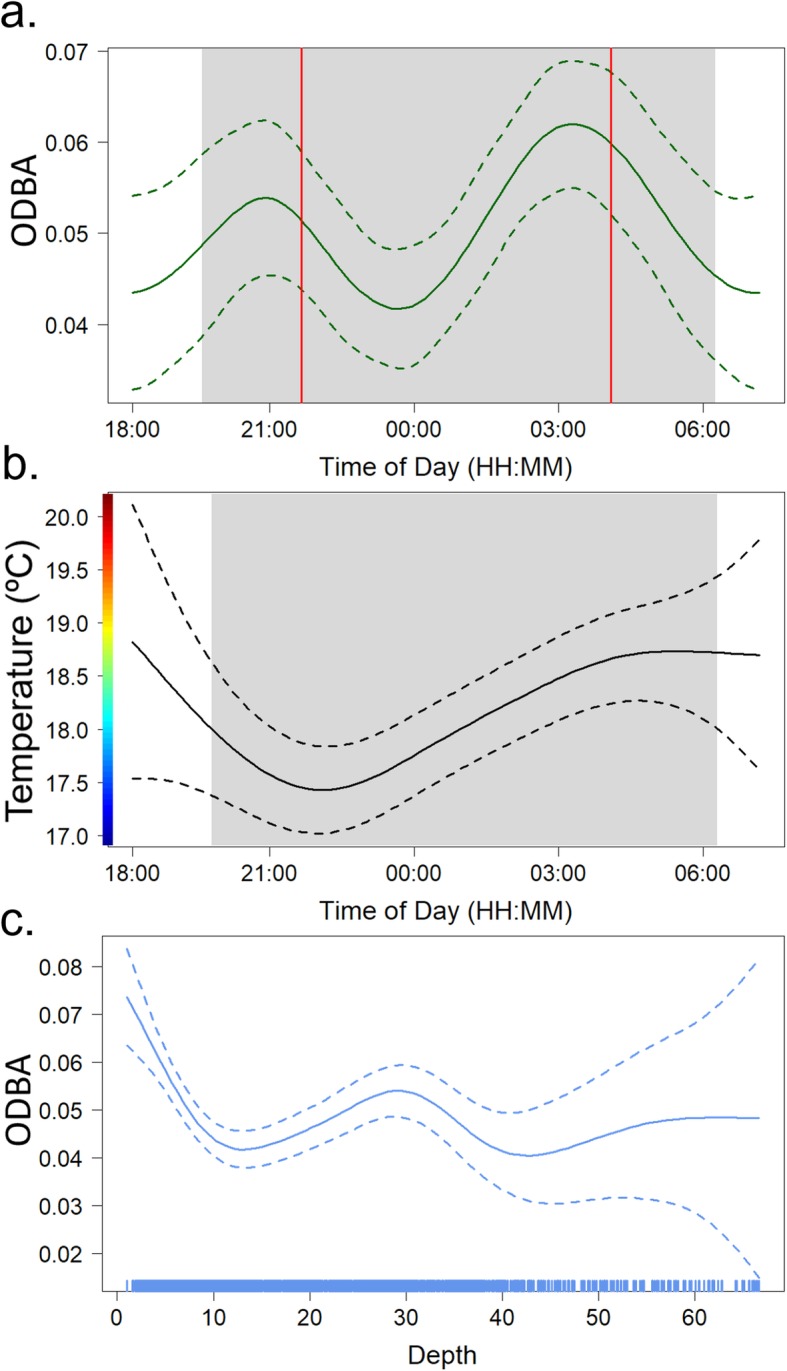
Hourly and environmental effects on California horn shark nighttime activity (ODBA) using general additive mixed models (GAMMs). Nighttime patterns in shark activity (**a**) and environmental temperature (**b**) calculated using 5 min means. Gray boxes denote median local sunset (19:43 PST) and local sunrise (06:14 PST) times from all active tracks. Red vertical lines (**a**) denote the median patch arrival (21:40 PST) and patch departure (04:03 PST) times from first passage time (FPT) analysis. **c** Horn shark activity was significantly influenced by what depth (m) the shark was at (Shark 06 removed from this analysis as an outlier). The blue rug in (**c**) indicates the spread of samples across the depths. Dashed lines (a, b, c) indicate the 95% confidence interval around each smooth term

Nighttime activity (ODBA) peaked around 21:00 and 03:30 (median, PST) (Fig. 5a), which coincided with the median times sharks would arrive and depart a patch (GAMM: *F* = 2.182, *p* = 0.001; Table 3). ODBA decreased as individuals arrived at a patch, yet the lowest levels of nighttime activity occurred around midnight. Temperature (as measured by the ADL) peaked around sunset as the water had warmed throughout the day while individuals rested. Temperature then decreased around 22:00 PST when most sharks were traversing through the deeper channels in between reefs (GAMM: *F* = 3.396, *p* = 0.01) (Fig. 5b, Table 3). After the initial decrease in temperature, temperature slowly increased over the remainder of the night until sunrise. Visually, there was no diel pattern between ODBA and temperature (Fig. 5a, b). Additionally, there was a significant relationship with depth and activity during the night (Shark 06 was removed from this analysis as an outlier). Activity peaked when depth was shallowest (1–10 m), decreased between 10 and 20 m, then peaked again around 30 m (GAMM: *F* = 6.83, *p* < 0.001; Fig. 5c, Table 3). Additionally, few sharks moved into depths greater than 40 m.

**Table 3:** General additive mixed models (GAMMs) fitted to explain the diel and environmental drivers of Overall Dynamic Body Acceleration (ODBA) of California horn sharks (*Heterodontus francisci*)

Model	Family	Sampling Window (min)	Resample Function	Correlation at lag 1	Degrees of freedom	F-statistic	*p* value of smoother
ODBA ~ s(Time of Day)	Gaussian	5	Mean	0.736	4.137	2.182	**0.0006**
ODBA ~ s(Temperature)	Gaussian	5	Mean	0.954	3.243	3.396	0.0108
ODBA ~ s(Depth)	Gaussian	5	Mean	0.731	6.217	6.895	**< 0.0001**

All GAMMs were performed on nighttime hours only (local sunset to sunrise) and included individual as a random factor. Significant fits are shown in bold; note that *p*-values of smoothers in GAM models fitted with *mgcv* are considered significant when *p* < 0.01 (Wood 2006). The correlation at lag 1 was determined by an autocorrelation function (ACF) and was included in the model to specify the autocorrelation term. Degrees of freedom are estimated by the *mgcv* package in R

## Discussion

This study combined high-resolution spatial movement and acceleration data to understand the fine-scale movement and activity patterns of a non-obligate ram ventilating elasmobranch. There was high individual variation across movement paths, regardless of where or when individuals were tagged, yet nocturnal activity and search modes (i.e., patch use) were consistent across individuals. All individuals rested in a shelter from sunrise to sunset, then departed their shelters at sunset in search of a resource patch. Acceleration data revealed that all individuals arrived and departed patches at similar times, regardless of size, sex, tagging location, or patch reef. Additionally, nighttime activity (ODBA) was strongly correlated (visually) to patch arrival and departure times, demonstrating bimodal peaks in activity representing the energetic costs of traversing through the relatively deeper (~ 30 m), cooler channels in between reefs. Once individuals were in a patch, there was an overall reduction in ODBA, likely because periods of inactivity were interspersed with periods of activity (i.e., resting, burst activity) and due to travel speeds decreasing within a patch [18, 19].

While it is unclear from our results what exactly was influencing the timing of patch behaviors, our activity classification within a patch (i.e., resting, episodic burst activity, moderate activity) allows us to hypothesize what behaviors are potentially driving horn shark movement patterns. Moderate activity within a patch may be indicative of prey or shelter searching, and episodic burst activity may be indicative of foraging or predator evasions, both of which could be energetically costly behaviors for these demersal sharks [80]. Therefore, the time spent inactive within a patch may offset the energetic costs associated with either traveling to a patch, or specific behaviors within a patch. From our results, we hypothesize patch use may be synced with the timing of nocturnal prey movements, assuming foraging is the goal of their ARS movements. Some nocturnal invertebrates exhibit varying degrees of diurnal vertical migrations (DVMs) [81, 82], and others (e.g., urchins) may take time to emerge from their protective daytime shelter habitats after sunset [83, 84]. Therefore, horn sharks may time their movements to locate a patch approximately 3 h after sunset as it optimizes their chances of more easily locating dense prey patches. To ultimately test this hypothesis, a more detailed study should consider quantifying the foraging acceleration signatures of these durophagous predators to determine the frequency and periodicity of feeding attempts during a given night [25, 26]. However, it is worth noting that our work was only done during the warmer season (Jun – Oct) and prey densities change seasonally. This may result in horn sharks exhibiting seasonally different movement patterns, so a future long-term telemetry study is warranted. This concept spans many taxa and should be taken into consideration for any study demonstrating ARS behaviors. For instance, Fauchald and Tveraa [85] conducted a study to quantify the spatial dynamics of the foraging areas of Antarctic petrels (*Thalossoica antarctica*). They found the petrels would use a hierarchical search strategy and move their large-scale search areas in response to seasonally varying densities and the spatial dynamics of Antarctic krill (*Euphausia superba*).

Visually, there was some overlap in ARS patches among individuals indicating an overlap of ‘search effort’, which may indicate areas where foraging success was greatest [15]. We found a significant relationship between the size of a patch and the time spent within a patch, yet the deviance explained was only approximately 10%. It is likely other factors we did not measure such as habitat type within a patch or foraging success may further explain time spent within a patch, thus increasing model deviance. It is important to note that quantifying the spatial heterogeneity of prey densities was not feasible during our study. However, the sizes of the patches observed (8–112 m radius) and the activity patterns within them may demonstrate if individuals had encountered either small densities of highly nutritional prey (via episodic burst activity), or larger densities of less nutritious prey (via consistent, moderate activity) [15, 20]. Temperate rocky reefs can be highly dynamic in regards to prey abundances and environmental conditions [86–88], further explaining the variability in movement paths we observed. ARS behaviors for horn sharks may emerge because individuals are being attracted to, or navigating to known predictable areas of prey, rather than to areas with higher prey densities [15, 17]. For instance, Southern elephant seals (*Mirounga leonina*) first use prior knowledge of predictable areas to drive large-scale movements, then secondarily use ARS to focus their search once in a profitable area [16]. Additionally, wandering albatross (Diomedeidae) only effectively use ARS within predictable and profitable environments [6]. Most of the patches (35%) we observed were on IR, the central most reef in our study site that experiences the greatest dynamic abiotic conditions and increased invertebrate diversity compared to the less exposed reefs in our study location. Therefore, regardless of where individuals were tagged, they may move towards this reef during the night because their probability of successful foraging may increase. Since active tracking methods produce short-term, fine-scale spatial information for a single individual at a time, it is unclear if horn sharks were travelling to patches together or if multiple individuals were attracted to a resource simultaneously [89]. However, during this study it was extremely rare (< 5 occurrences, observed while diving) to find horn sharks resting together in the same den during the day, indicating they are probably more solitary as previous studies suggest [51, 52].

The overlapping search effort of the patches occurred on a few key reefs, suggesting those reefs are potentially profitable habitat and ‘activity hotspots’ [15, 42]. It is important to note that while tagging was done opportunistically at different reefs, there was no consistent pattern between location of tagging and location of patches. In other words, tagging individuals at one reef did not indicate they would use patches either on that reef or a different reef. Within a marine community, spatial heterogeneity of habitat type (i.e., boulder, cobble, sand) can lead to differences in prey type and availability [3]. Therefore, individual prey preferences may be driving the degree of patch spatial overlap among individuals. Horn sharks are suction feeders their entire lives, with durophagy occurring at adolescence [90–92]. After durophagy occurs, adults have the potential to become prey specialists as they mature, thereby driving conspecific niche specialization [52, 54, 93]. Therefore, it is important to note that while sharks tracked were considered mature (range: 59–89 cm TL), the age of an individual may also play a role in prey preferences and feeding abilities. Since we only tagged adults due to the size of our tag package, prey specialization may partition horn sharks into using different areas within their home range, which may explain the individual variation in the sizes and locations of patches. Additionally, of the 12 sharks we tagged for two nighttime periods (48 h tracking duration), only one shark demonstrated repeated movement paths from the first night on the second night. For any taxa exhibiting ARS movements, there are likely mechanisms in place regulating the profitability of revisiting a patch over time. If an animal revisits a patch repeatedly, there could be negative consequences for the stability of prey populations in those areas [91]. Therefore, a foraging animal likely relies on a combination of innate and learned behaviors, as well as the ability to use predictable information about prey abundances and environmental conditions to ultimately determine what might be the most efficient movement strategy [6, 16, 42, 94].

Although we hypothesize foraging efforts and patchy prey distributions are the likely drivers of patch use, other behaviors could have equally resulted in the observed ARS movement paths. For instance, while patches characterized as episodic burst activity (48% of active patches) may have been indicative of successful foraging attempts, predator evasions could have also produced the ODBA signatures observed in these patches [25]. Horn sharks are relatively smaller sharks (TL < 122 cm [95];) and past studies have noted smaller sharks are more vulnerable to predation [96, 97]. Therefore, under certain conditions, predator abundances could be high enough to influence horn shark movement and activity patterns. The risk of predation can structure how animals use their landscapes [4, 98] by triggering food versus risk trade-offs that can influence movement and activity patterns [99, 100]. A classic example of this is the reintroduction of wolves in Yellowstone National Park where the addition of wolves correlated with changes in elk reproductive fitness and a decrease in elk populations [4, 99, 101]. Additionally, in the marine environment, Heupel and Heuter [89] concluded that juvenile blacktip sharks (*Carcharhinus limbatus*), were using less productive habitats to avoid predators. Therefore, determining the ultimate motivation behind ARS behaviors, although difficult, is critical to correctly interpreting movement path structures.

Surprisingly, our work found no correlation between ODBA and water temperature for these ectothermic elasmobranchs. While average nighttime activity was highest when individuals were traveling in relatively deeper, cooler channels in between reefs, it was not apparent from these results that horn sharks were purposely seeking an energetic advantage of moving through cooler water to minimize activity costs [38, 102]. Since horn sharks swim no more than 2 m off the seafloor [52], they may have been traversing through those habitats simply because that is the only way to travel to their intended patch reef or shelter. Additionally, increased ODBA in these relatively deeper, cooler habitats may have been due to increased swimming speeds (relative to speeds within patches) because they are more vulnerable to predation when they are in unprotected habitats, particularly if their internal body temperatures may be too cool to efficiently evade predators [38]. Our results are comparable to other studies for ectothermic elasmobranchs that found environmental temperature did not seem to drive movement and activity patterns. For example, freshwater sawfish (*Pristis pristis*) increase activity during crepuscular and nocturnal periods, regardless of thermal environment [25]. While sawfish were found to be resting in cooler water, there was no clear pattern between activity and temperature, indicating temperature did not play a significant role in driving movement patterns [25]. Additionally, body temperatures of gray reef sharks (*Carcharhinus amblyrhynchos*) had an insignificant effect on routine metabolic rates (RMRs) at the diel scale, and instead diel changes in RMRs were driven by swim speeds [21].

Luongo and Lowe [103] determined horn sharks have a relatively low, resting (standard metabolic rate; SMR), metabolic Q_10_ (2.01), demonstrating that they are relatively thermally insensitive. This may explain why we saw no clear pattern between activity and temperature, yet we did see clear patterns in diel activity. However, it is important to note Luongo and Lowe [103] additionally found that horn sharks minimum energetic costs (i.e., SMR) increased by 23% in warmer temperatures (> 20 °C), indicating these sharks physiologically perform better in cooler conditions (< 20 °C) and likely exhibit some compensatory behaviors when environmental conditions become more energetically costly (i.e., warmer). Therefore, further studies on the influence of environmental temperature on the movement and activity patterns of horn sharks are warranted, specifically in regards to how they may be selecting temperatures compared to what is available within their home range [25, 35]. Furthermore, this would be particularly of interest in regards to increasing temperatures associated with global climate change, and how rocky reef communities may be affected.

## Conclusions

This study provides one of the largest fine-scale, high-resolution paired data sets available for an elasmobranchmovement ecology study. We demonstrate that horn sharks exhibited ARS movement patterns and displayed various activity patterns within ARS patches. Our work found that horn shark movements and activity patterns are not reliant upon environmental temperature, indicating they may instead focus their movements by traveling to reefs known to have profitable and predictable patches. As nocturnal, hard prey (e.g., urchins) specialists known to be annual residents in rocky reef communities, horn sharks may be ecologically important regulators of lower trophic level invertebrates, potentially responsible for regulating kelp bed community structure [88, 104–107]. Our study highlights the importance of gathering fine-scale, high-resolution information of any taxa to better understand daily movement decisions to enhance knowledge of community structure and overall ecosystem function.

## Supplementary information

**Additional file 1. **Acceleration summaries for actively tracked California horn sharks (*Heterodontus francisci*). Mean values for ODBA (Overall Dynamic Body Acceleration), temperature (°C), and depth (m) for each 24 h period for each California horn shark tracked.

**Additional file 2. **Example of *k*-means cluster analysis. Example of how *k*-means clusters were derived from the acceleration ethogram in IgorPro using Ethographer (Sakamoto et al. 2009). Clusters were then used to quantify if California horn sharks were resting or active.

**Additional file 3.** Diel comparisons of Brownian bridge kernel utilization distributions (BBKUDs). Diel comparisons of Brownian bridge kernel utilization distributions (BBKUDs) for California horn sharks for both 95% (daily) and 50% (core) activity spaces estimates.

## Data Availability

Data used in this study is available upon reasonable request to the corresponding author.

## Abbreviations

ADL: Acceleration data logger
ARS: Area restricted search
BFC: Big Fisherman’s Cove
BR: Bird Rock
CG: Campgrounds
CGM: Campground Moorings
dB: decibels
FPT: First passage time
GAMM: General Additive Mixed Model
IR: Isthmus Reef
LME: Linear mixed effects model
MCP: Minimum Convex Polygon
ODBA: Overall Dynamic Body Acceleration
TL: Total length

## References

[1] Hill S, Burrows M, Hughes R (2003). The efficiency of adaptive search tactics for different prey distribution patterns: a simulation model based on the behaviour of juvenile plaice. J Fish Biol.

[2] Sims DW, Witt MJ, Richardson AJ, Southall EJ, Metcalfe JD (2006). Encounter success of free-ranging marine predator movements across a dynamic prey landscape. Proc R Soc B Biol Sci.

[3] Shepard ELC, Wilson RP, Quintana F, Gómez Laich A, Liebsch N, Albareda DA, Halsey LG, Gleiss A, Morgan DT, Myers AE, Newman C, McDonald DW (2008). Identification of animal movement patterns using tri-axial accelerometry. Endangered Species Research.

[4] Gallagher AJ, Creel S, Wilson RP, Cooke SJ (2017). Energy landscapes and the landscape of fear. Trends Ecol Evol.

[5] Zollner PA, Lima SL (1999). Search strategies for landscape-level interpatch movements. Ecology.

[6] Weimerskirch H, Pinaud D, Pawlowski F, Bost C-A (2007). Does prey capture induce area-restricted search? A fine-scale study using GPS in a marine predator, the wandering albatross. Am Nat.

[7] Nams VO (2006). Detecting oriented movement of animals. Anim Behav.

[8] Bartumeus F, da Luz MGE, Viswanathan GM, Catalan J (2005). Animal search strategies: a quantitative random-walk analysis. Ecology.

[9] Reynolds AM, Rhodes CJ (2009). The Lévy flight paradigm: random search patterns and mechanisms. Ecology.

[10] Benhamou S (1992). Efficiency of area-concentrated searching behaviour in a continuous patchy environment. J Theor Biol.

[11] Papastamatiou YP, DeSalles PA, McCauley DJ (2012). Area-restricted searching by manta rays and their response to spatial scale in lagoon habitats. Mar Ecol Prog Ser.

[12] Kareiva P, Odell G (1987). Swarms of predators exhibit" preytaxis" if individual predators use area-restricted search. Am Nat.

[13] Biesinger Z, Haefner JW (2005). Proximate cues for predator searching: a quantitative analysis of hunger and encounter rate in the ladybird beetle, *Coccinella septempunctata*. Anim Behav.

[14] Fauchald P, Tveraa T (2003). Using first-passage time in the analysis of area-restricted search and habitat selection. Ecology.

[15] Bailey H, Thompson P (2006). Quantitative analysis of bottlenose dolphin movement patterns and their relationship with foraging. J Anim Ecol.

[16] Thums M, Bradshaw CJ, Hindell MA (2011). In situ measures of foraging success and prey encounter reveal marine habitat-dependent search strategies. Ecology.

[17] Begg GS, Reid JB (1997). Spatial variation in seabird density at a shallow sea tidal mixing front in the Irish Sea. ICES J Mar Sci.

[18] Hill S, Burrows M, Hughes R (2000). Increased turning per unit distance as an area-restricted search mechanism in a pause-travel predator, juvenile plaice, foraging for buried bivalves. J Fish Biol.

[19] Newlands NK, Lutcavage ME, Pitcher TJ (2004). Analysis of foraging movements of Atlantic bluefin tuna (*Thunnus thynnus*): individuals switch between two modes of search behaviour. Popul Ecol.

[20] Fauchald P (2009). Spatial interaction between seabirds and prey: review and synthesis. Mar Ecol Prog Ser.

[21] Papastamatiou YP, Watanabe YY, Demšar U, Leos-Barajas V, Bradley D, Langrock R, Weng K, Lowe CG, Friedlander AM, Caselle JE (2018). Activity seascapes highlight central place foraging strategies in marine predators that never stop swimming. Mov Ecol.

[22] Nakamura I, Watanabe YY, Papastamatiou YP, Sato K, Meyer CG (2011). Yo-yo vertical movements suggest a foraging strategy for tiger sharks *Galeocerdo cuvier*. Mar Ecol Prog Ser.

[23] Papastamatiou YP, Cartamil DP, Lowe CG, Meyer CG, Wetherbee BM, Holland KN (2011). Scales of orientation, directed walks and movement path structure in sharks. J Anim Ecol..

[24] Papastamatiou YP, Meyer CG, Carvalho F, Dale JJ, Hutchinson MR, Holland KN (2013). Telemetry and random-walk models reveal complex patterns of partial migration in a large marine predator. Ecology..

[25] Gleiss AC, Morgan DL, Whitty JM, Keleher JJ, Fossette S, Hays GC (2017). Are vertical migrations driven by circadian behaviour? Decoupling of activity and depth use in a large riverine elasmobranch, the freshwater sawfish (*Pristis pristis*). Hydrobiologia.

[26] Whitney NM, Pratt HL, Pratt TC, Carrier JC (2010). Identifying shark mating behaviour using three-dimensional acceleration loggers. Endanger Species Res.

[27] Meese EN, Lowe CG (2019). Finding a resting place: how environmental conditions influence the habitat selection of resting batoids. SCAS Bulletin.

[28] Angilletta Michael J. (2009). Thermoregulation. Thermal Adaptation.

[29] Gilchrist GW, Storey KB, Storey J (2000). The evolution of thermal sensitivity in changing environments*.* Environmental stressors and gene response.

[30] Schmidt-Nielsen K. Animal Physiology: Adaptation and Environment. United Kingdom: Cambridge University Press; 1997.

[31] Brett JR (1971). Energetic responses of salmon to temperature. A study of some thermal relations in the physiology and freshwater ecology of sockeye salmon (*Oncorhynchus nerkd*). Am Zool.

[32] Bernal D, Lowe CG. Fish Physiology: Field Physiology of Elasmobranch Fishes. Fish Physiology: Physiology of Elasmobranch Fishes: Structure and Interaction with Environment, ed. A.P.F. R.E. Shadwick, C.J. Brauner. Vol. 34A. USA: Academic Press. 2015.

[33] Somero GN (1995). Proteins and temperature. Annu Rev Physiol.

[34] Gillooly JF, Brown JH, West GB, Savage VM, Charnov EL (2001). Effects of size and temperature on metabolic rate. Science.

[35] Hight BV, Lowe CG (2007). Elevated body temperatures of adult female leopard sharks, *Triakis semifasciata*, while aggregating in shallow nearshore embayments: evidence for behavioral thermoregulation?. J Exp Mar Biol Ecol.

[36] Matern SA, Cech JJ, Hopkins TE (2000). Diel movements of bat rays, *Myliobatis californica*, in Tomales Bay, California: evidence for behavioral thermoregulation?. Environ Biol Fish.

[37] Wallman HL, Bennett WA (2006). Effects of parturition and feeding on thermal preference of Atlantic stingray, *Dasyatis sabina* (Lesueur). Environ Biol Fish.

[38] Papastamatiou YP, Watanabe YY, Bradley D, Dee LE, Weng K, Lowe CG, Caselle JE (2015). Drivers of daily routines in an ectothermic marine predator: hunt warm, rest warmer?. PLoS One.

[39] Simpfendorfer CA, Heupel MR (2004). Assessing habitat use and movement*.* Biology of sharks and their relatives.

[40] Sims DW (2010). Tracking and analysis techniques for understanding free-ranging shark movements and behavior*.* Sharks and their relatives II: biodiversity, adaptive physiology, and conservation.

[41] Hammerschlag N, Gallagher A, Lazarre D (2011). A review of shark satellite tagging studies. J Exp Mar Biol Ecol.

[42] Papastamatiou YP, Lowe CG (2012). An analytical and hypothesis-driven approach to elasmobranch movement studies. J Fish Biol.

[43] Whitney NM, Papastamatiou YP, Gleiss AC, Carrier J, Musick J, Heithaus M, Carrier JC, Musick JA, Heithaus MR (2012). Integrative multi-sensor tagging: emerging techniques to link elasmobranch behavior, physiology and ecology*.* Sharks and Their Relatives.

[44] Andrzejaczek S, Gleiss AC, Lear KO, Pattiaratchi CB, Chapple T, Meekan M (2019). Biologging tags reveal links between fine-scale horizontal and vertical movement behaviours in tiger sharks (*Galeocerdo cuvier*). Front Mar Sci.

[45] Gleiss AC, Dale JJ, Holland KN, Wilson RP (2010). Accelerating estimates of activity-specific metabolic rate in fishes: testing the applicability of acceleration data-loggers. J Exp Mar Biol Ecol.

[46] Wilson RP, White CR, Quintana F, Halsey LG, Liebsch N, Martin GR, Butler PJ (2006). Moving towards acceleration for estimates of activity-specific metabolic rate in free-living animals: the case of the cormorant. J Anim Ecol.

[47] Gleiss AC, Norman B, Liebsch N, Francis C, Wilson RP (2009). A new prospect for tagging large free-swimming sharks with motion-sensitive data-loggers. Fish Res.

[48] Wilson RP, Shepard EL, Liebsch N (2008). Prying into the intimate details of animal lives: use of a daily diary on animals. Endanger Species Res.

[49] Brownscombe JW, Cooke SJ, Danylchuk AJ (2017). Spatiotemporal drivers of energy expenditure in a coastal marine fish. Oecologia.

[50] Shepard EL, Wilson RP, Rees WG, Grundy E, Lambertucci SA, Vosper SB (2013). Energy landscapes shape animal movement ecology. Am Nat.

[51] Nelson Donald R., Johnson Richard H. (1970). Diel Activity Rhythms in the Nocturnal, Bottom-Dwelling Sharks, Heterodontus francisci and Cephaloscyllium ventriosum. Copeia.

[52] Strong WR (1989). Behavioral ecology of horn sharks, Heterodontus francisci, at Santa Catalina Island, California, with emphasis on patterns of space utilization.

[53] Vaudo JJ, Heithaus MR (2009). Spatiotemporal variability in a sandflat elasmobranch fauna in Shark Bay, Australia. Mar Biol.

[54] Segura-Zarzosa JC, Abitia-Cárdenas LA, Galván-Magaña F (1997). Observaciones sobre la alimentación del tiburón *Heteredontus francisci* (Girard, 1854)(Chondrichthyes: Heterodontidae), en Laguna de San Ignacio, Baja California Sur, México. Cienc.

[55] Ahr B, Farris M, Lowe CG (2015). Habitat selection and utilization of white croaker (*Genyonemus lineatus*) in the Los Angeles and Long Beach harbors and the development of predictive habitat use models. Mar Environ Res.

[56] Lowe CG, Topping DT, Cartamil DP, Papastamatiou YP (2003). Movement patterns, home range, and habitat utilization of adult kelp bass *Paralabrax clathratus* in a temperate no-take marine reserve. Mar Ecol Prog Ser.

[57] McKinzie MK, Jarvis ET, Lowe CG (2014). Fine-scale horizontal and vertical movement of barred sand bass, *Paralabrax nebulifer*, during spawning and non-spawning seasons. Fish Res.

[58] Agafonkin V, Thieurmel B (2017). suncalc: Compute sun position, sunlight phases, moon position, and lunar phase. R package version 0.3.

[59] Calenge Clément (2006). The package “adehabitat” for the R software: A tool for the analysis of space and habitat use by animals. Ecological Modelling.

[60] Horne JS, Garton EO, Krone SM, Lewis JS (2007). Analyzing animal movements using Brownian bridges. Ecology.

[61] Heupel MR, Simpfendorfer CA, Hueter RE (2004). Estimation of shark home ranges using passive monitoring techniques. Environ Biol Fish.

[62] Bates D, Mächler M, Bolker B, Walker S (2014). Fitting linear mixed-effects models using lme4. arXiv preprint arXiv:1406.5823.

[63] Bates D, Maechler M, Bolker B, Walker S (2014). lme4: Linear mixed-effects models using Eigen and S4. R package version 1.1-7.

[64] Fox J, Weisberg S, Adler D, Bates D, Baud-Bovy G, Ellison S, Firth D, Friendly M, Gorjanc G, Graves S (2012). Package ‘car’.

[65] Kuznetsova A, Brockhoff PB, Christensen RHB. lmerTest package: tests in linear mixed effects models. J Stat Softw. 2017;82:1-26.

[66] Calenge C (2011). Analysis of animal movements in R: the adehabitatLT package.

[67] Barraquand F, Benhamou S (2008). Animal movements in heterogeneous landscapes: identifying profitable places and homogeneous movement bouts. Ecology.

[68] Lavielle M (2005). Using penalized contrasts for the change-point problem. Signal Process.

[69] Sakamoto KQ, Sato K, Ishizuka M, Watanuki Y, Takahashi A, Daunt F, Wanless S (2009). Can ethograms be automatically generated using body acceleration data from free-ranging birds?. PLoS One.

[70] Shepard EL, Wilson RP, Halsey LG, Quintana F, Laich AG, Gleiss AC, Adrian C, Liebsch N, Myers AE, Norman B (2008). Derivation of body motion via appropriate smoothing of acceleration data. Aquat Biol.

[71] Halsey LG, Shepard EL, Hulston CJ, Venables MC, White CR, Jeukendrup AE, Wilson RP (2008). Acceleration versus heart rate for estimating energy expenditure and speed during locomotion in animals: tests with an easy model species, *Homo sapiens*. Zoology.

[72] Gleiss AC, Wright S, Liebsch N, Wilson RP, Norman B (2013). Contrasting diel patterns in vertical movement and locomotor activity of whale sharks at Ningaloo reef. Mar Biol.

[73] Lear KO, Whitney NM, Brewster LR, Morris JJ, Hueter RE, Gleiss AC, Adrian C (2017). Correlations of metabolic rate and body acceleration in three species of coastal sharks under contrasting temperature regimes. J Exp Biol.

[74] Tanaka H, Takagi Y, Naito Y (2001). Swimming speeds and buoyancy compensation of migrating adult chum salmon *Oncorhynchus keta* revealed by speed/depth/acceleration data logger. J Exp Biol.

[75] Watanuki Y, Takahashi A, Daunt F, Wanless S, Harris M, Sato K, Naito Y (2005). Regulation of stroke and glide in a foot-propelled avian diver. J Exp Biol.

[76] Hart KM, White CF, Iverson AR, Whitney N (2016). Trading shallow safety for deep sleep: juvenile green turtles select deeper resting sites as they grow. Endanger Species Res.

[77] Gleiss AC, Schallert RJ, Dale JJ, Wilson SG, Block BA (2019). Direct measurement of swimming and diving kinematics of giant Atlantic bluefin tuna (*Thunnus thynnus*). R Soc Open Sci.

[78] Wood SN. Generalized additive models: an introduction with R. Boca Raton: Chapman and Hall/CRC; 2017.

[79] Zuur A, Ieno EN, Walker N, Saveliev AA, Smith GM. Mixed effects models and extensions in ecology with R. New York: Springer Science & Business Media; 2009.

[80] Gleiss AC, Wilson RP, Shepard EL (2011). Making overall dynamic body acceleration work: on the theory of acceleration as a proxy for energy expenditure. Methods Ecol Evol.

[81] Van Noord JE, Dorval E (2017). Oceanographic influences on the distribution and relative abundance of market squid paralarvae (*Doryteuthis opalescens*) off the southern and Central California coast. Mar Ecol.

[82] Hays Graeme C. (2003). A review of the adaptive significance and ecosystem consequences of zooplankton diel vertical migrations. Migrations and Dispersal of Marine Organisms.

[83] Harrold C, Reed DC (1985). Food availability, sea urchin grazing, and kelp forest community structure. Ecology.

[84] Schiel DR, Foster MS. The biology and ecology of giant kelp forests. Oakland: Univ of California Press; 2015.

[85] Fauchald P, Tveraa T (2006). Hierarchical patch dynamics and animal movement pattern. Oecologia.

[86] Dayton PK, Tegner MJ, Parnell PE, Edwards PB (1992). Temporal and spatial patterns of disturbance and recovery in a kelp forest community. Ecol Monogr.

[87] Dayton PK, Tegner MJ (1984). Catastrophic storms, El Niño, and patch stability in a southern California kelp community. Science.

[88] Tegner MJ, Dayton PK (2000). Ecosystem effects of fishing in kelp forest communities. ICES J Mar Sci.

[89] Heupel MR, Hueter RE (2002). Importance of prey density in relation to the movement patterns of juvenile blacktip sharks (*Carcharhinus limbatus*) within a coastal nursery area. Mar Freshw Res.

[90] Summers AP, Ketcham RA, Rowe T (2004). Structure and function of the horn shark (*Heterodontus francisci*) cranium through ontogeny: development of a hard prey specialist. J Morphol.

[91] Edmonds MA, Motta PJ, Hueter RE (2001). Food capture kinematics of the suction feeding horn shark, Heterodontus francisci. Environ Biol Fish.

[92] Wu E (1988). The functional morphology of suction feeding in the horn shark (Heterodontiformes) and the whitespotted bamboo shark (Orectolobiformes). AES annual meeting abstracts.

[93] Huber DR, Eason TG, Hueter RE, Motta PJ (2005). Analysis of the bite force and mechanical design of the feeding mechanism of the durophagous horn shark *Heterodontus francisci*. J Exp Biol.

[94] Lowe CG, Bray RN (2006). Fish movement and activity patterns.

[95] Compagno LJ. Sharks of the world: an annotated and illustrated catalogue of shark species known to date, vol. 2. Rome: Food & Agriculture Organization of United Nations; 2001.

[96] Heupel MR, Simpfendorfer CA (2002). Estimation of mortality of juvenile blacktip sharks, within a nursery area using telemetry data. Canadian Journal of Fisheries and Aquatic Sciences.

[97] Lowe C (2001). Metabolic rates of juvenile scalloped hammerhead sharks (Sphyrna lewini). Mar Biol.

[98] Laundré JW, Hernández L, Altendorf KB (2001). Wolves, elk, and bison: reestablishing the" landscape of fear" in Yellowstone National Park, USA. Can J Zool.

[99] Creel S, Winnie JA, Christianson D (2009). Glucocorticoid stress hormones and the effect of predation risk on elk reproduction. Proc Natl Acad Sci.

[100] Christianson D, Creel S (2010). A nutritionally mediated risk effect of wolves on elk. Ecology.

[101] Fortin D, Beyer HL, Boyce MS, Smith DW, Duchesne T, Mao JS (2005). Wolves influence elk movements: behavior shapes a trophic cascade in Yellowstone National Park. Ecology.

[102] Di Santo V, Bennett WA (2011). Effect of rapid temperature change on resting routine metabolic rates of two benthic elasmobranchs. Fish Physiol Biochem.

[103] Luongo SM, Lowe CG (2018). Seasonally acclimated metabolic Q 10 of the California horn shark, *Heterodontus francisci*. J Exp Mar Biol Ecol.

[104] Steneck RS, Graham MH, Bourque BJ, Corbett D, Erlandson JM, Estes JA, Tegner MJ (2002). Kelp forest ecosystems: biodiversity, stability, resilience and future. Environ Conserv.

[105] Rasher DB, Hoey AS, Hay ME (2017). Cascading predator effects in a Fijian coral reef ecosystem. Sci Rep.

[106] Harley CD (2011). Climate change, keystone predation, and biodiversity loss. Science.

[107] Borer E, Seabloom E, Shurin J, Anderson K, Blanchette C, Broitman B, Cooper S, Halpern B (2005). What determines the strength of a trophic cascade?. Ecology.

