# Supplementary material for: Active acoustic telemetry tracking and tri-axial accelerometers reveal fine-scale movement strategies of a non-obligate ram ventilator
Source: Mov Ecol. 2020 Feb 10;8:8. doi: 10.1186/s40462-020-0191-3 (PMC7011439; doi:10.1186/s40462-020-0191-3)
Supplement: Supplementary file 1 — Additional file 1. Acceleration summaries for actively tracked California horn sharks (Heterodontus francisci). Mean values for ODBA (Overall Dynamic Body Acceleration), temperature (°C), and depth (m) for each 24 h period for each California horn shark tracked. [file 40462_2020_191_MOESM1_ESM.docx]

Additional file 1. Acceleration data logger summaries for each 24 hr tracking period for all California horn sharks (*Heterodontus francisci*) continuously, actively tracked.


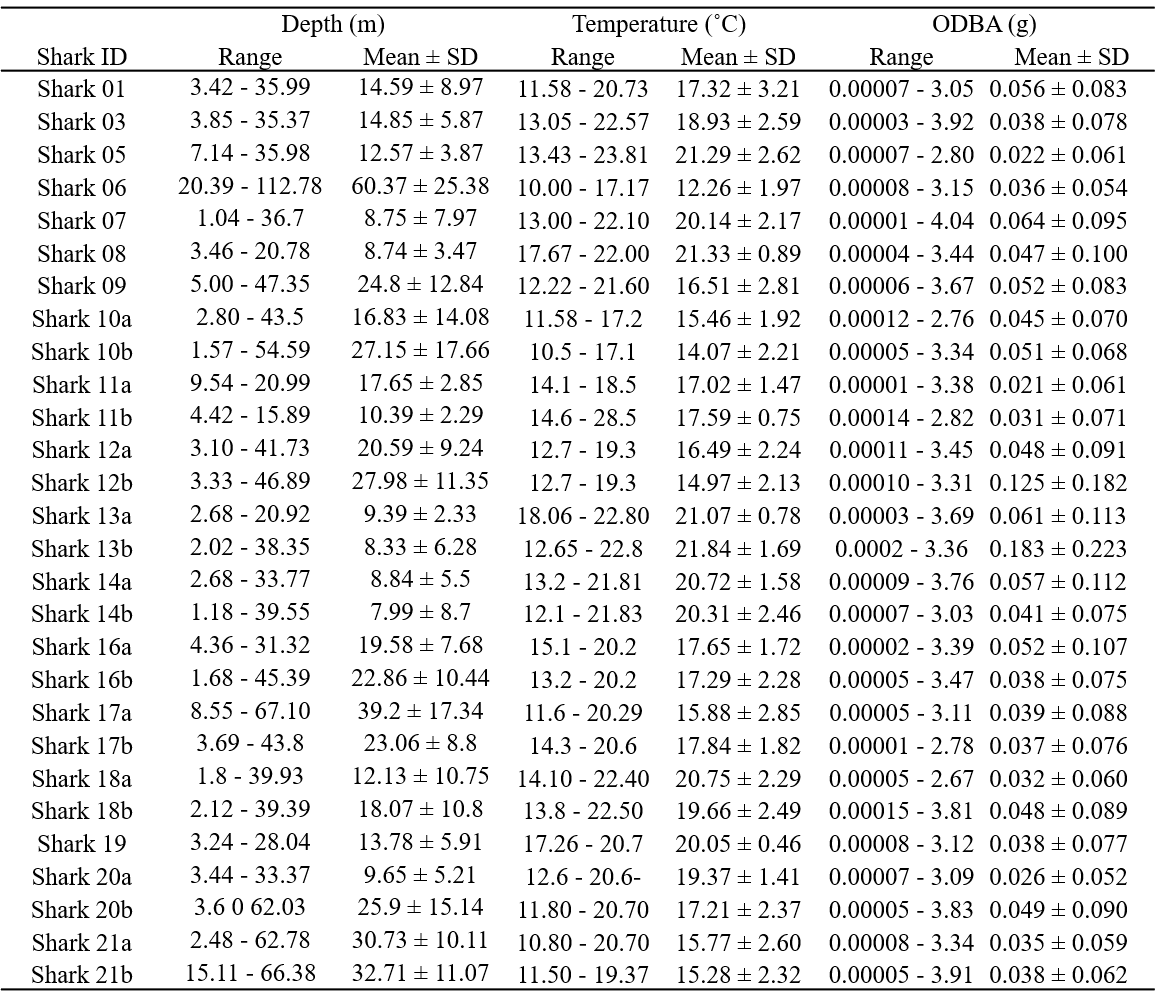


Note: Sharks tracked for > 24 h (Shark 10 – 21) are broken down into each nighttime tracking period, where “a” designates the first night, and “b” designates the second night. Sharks 1 – 9 were only tracked for 24 h, resulting in a single nighttime period.
