# Supplementary material for: Active acoustic telemetry tracking and tri-axial accelerometers reveal fine-scale movement strategies of a non-obligate ram ventilator
Source: Mov Ecol. 2020 Feb 10;8:8. doi: 10.1186/s40462-020-0191-3 (PMC7011439; doi:10.1186/s40462-020-0191-3)
Supplement: Supplementary file 2 — Additional file 2. Example of k-means cluster analysis. Example of how k-means clusters were derived from the acceleration ethogram in IgorPro using Ethographer (Sakamoto et al. 2009). Clusters were then used to quantify if California horn sharks were resting or active. [file 40462_2020_191_MOESM2_ESM.docx]

**Additional file 2**. Example of *k*-means cluster analysis to quantify resting and active behavior of California horn sharks (*Heterodontus francisci*).


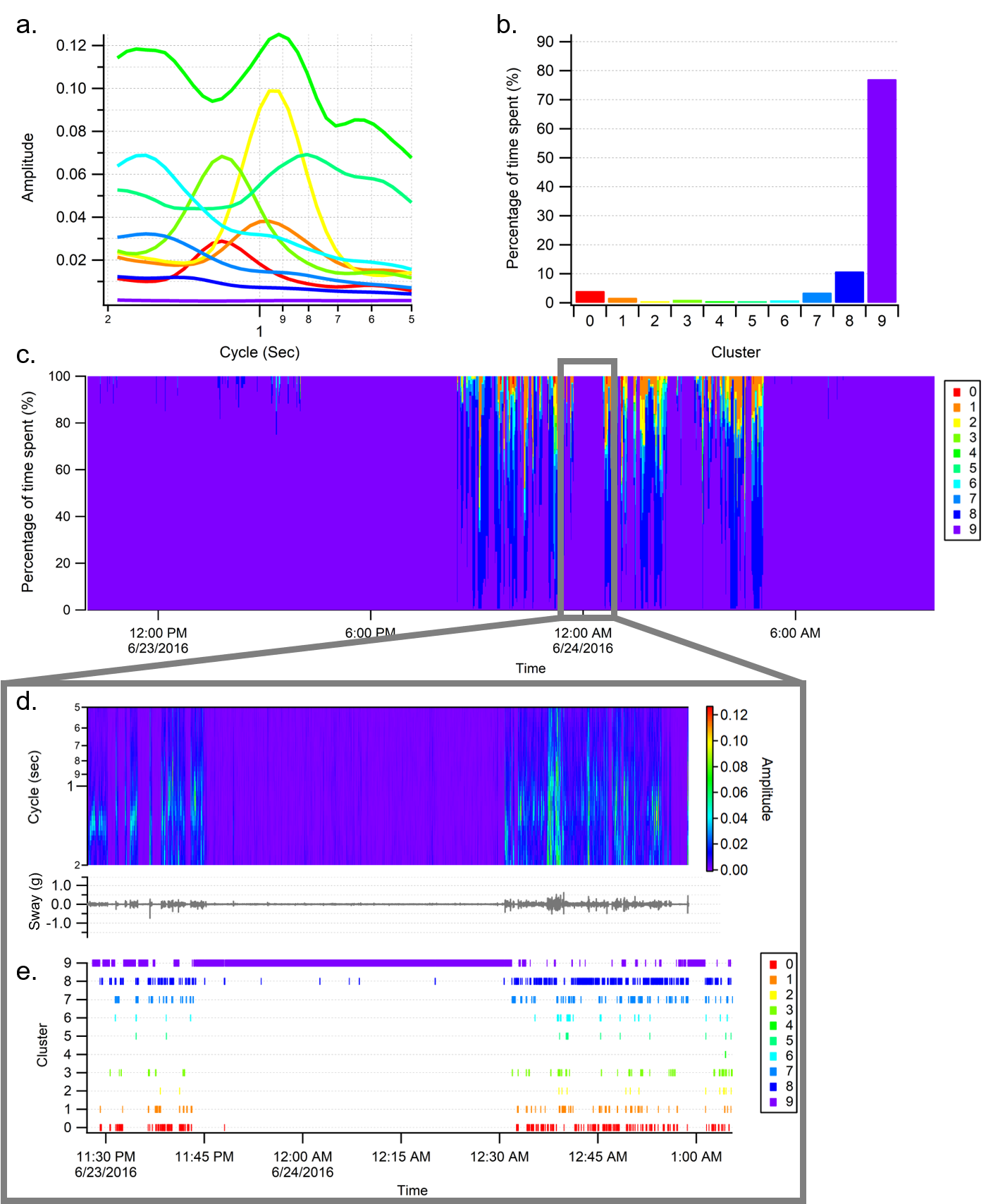


a) Signal strength and amplitude of each cluster formed by the *k*-means analysis. b) Percentage of time an individual spent exhibiting the behavior of each identified cluster. c) Percentage of time an individual spent exhibiting the behavior of each identified cluster shown over a 24 h diel cycle. d) A zoomed in portion of the acceleration ethogram used to create the *k*-means clusters. e) A zoomed in portion of the clusters correlated with the acceleration ethogram to demonstrate how the clusters are derived from the acceleration ethogram.
