# Supplementary material for: Active acoustic telemetry tracking and tri-axial accelerometers reveal fine-scale movement strategies of a non-obligate ram ventilator
Source: Mov Ecol. 2020 Feb 10;8:8. doi: 10.1186/s40462-020-0191-3 (PMC7011439; doi:10.1186/s40462-020-0191-3)
Supplement: Supplementary file 3 — Additional file 3. Diel comparisons of Brownian bridge kernel utilization distributions (BBKUDs). Diel comparisons of Brownian bridge kernel utilization distributions (BBKUDs) for California horn sharks for both 95% (daily) and 50% (core) activity spaces estimates. [file 40462_2020_191_MOESM3_ESM.docx]

**Additional file 3** Diel comparison of Brownian bridge kernel utilization distributions (BBKUDs) for California horn sharks (*Heterodontus francisci*).


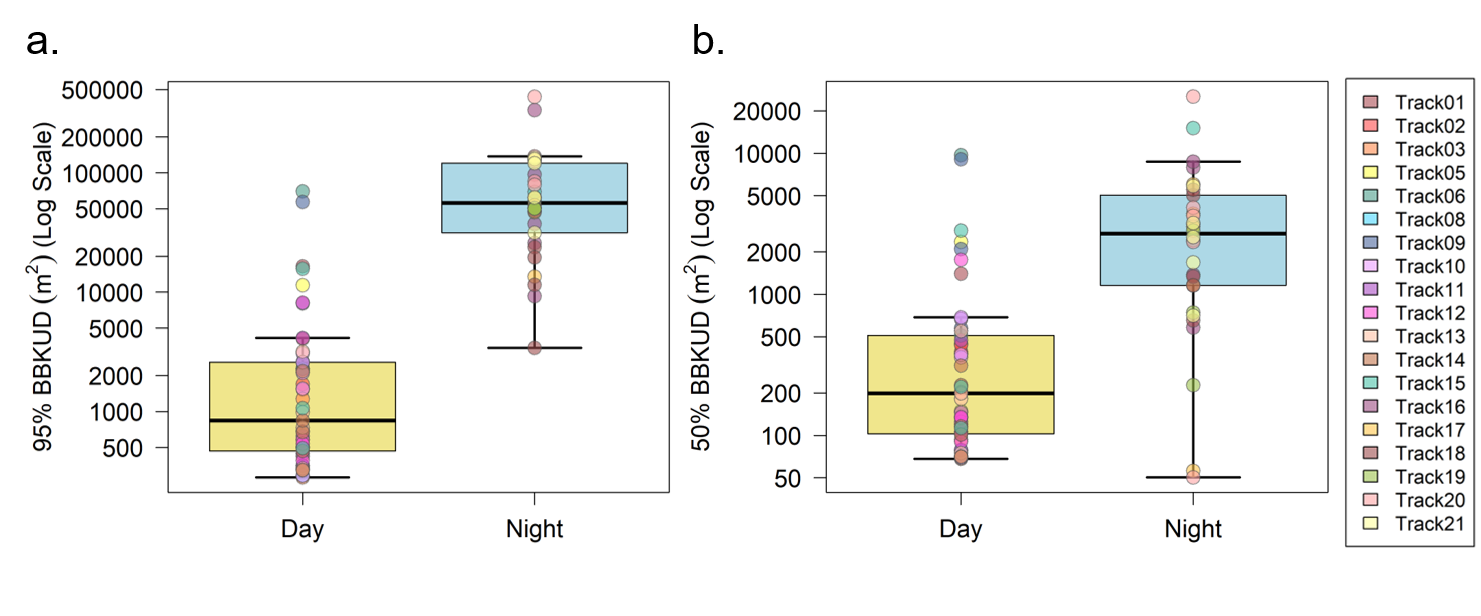


a) Day and night 95% BBKUDs for California horn sharks to represent daily activity space. b) Day and night 50% BBKUDs for California horn sharks to represent core activity space. Points are colored by individual shark and separated out by 24 h diel cycles tracked. Note the log-scale on the y-axis for visual representation.
